# Weight Estimation Tool for Children Aged 6 to 59 Months in Limited-Resource Settings

**DOI:** 10.1371/journal.pone.0159260

**Published:** 2016-08-16

**Authors:** Mark E. Ralston, Mark A. Myatt

**Affiliations:** 1 Department of Pediatrics, Uniformed Services University of the Health Sciences, Bethesda, Maryland, United States of America; 2 Brixton Health, Llawryglyn, Powys, Wales, United Kingdom; TNO, NETHERLANDS

## Abstract

**Importance:**

A simple, reliable anthropometric tool for rapid estimation of weight in children would be useful in limited-resource settings where current weight estimation tools are not uniformly reliable, nearly all global under-five mortality occurs, severe acute malnutrition is a significant contributor in approximately one-third of under-five mortality, and a weight scale may not be immediately available in emergencies to first-response providers.

**Objective:**

To determine the accuracy and precision of mid-upper arm circumference (MUAC) and height as weight estimation tools in children under five years of age in low-to-middle income countries.

**Design:**

This was a retrospective observational study. Data were collected in 560 nutritional surveys during 1992–2006 using a modified Expanded Program of Immunization two-stage cluster sample design.

**Setting:**

Locations with high prevalence of acute and chronic malnutrition.

**Participants:**

A total of 453,990 children met inclusion criteria (age 6–59 months; weight ≤ 25 kg; MUAC 80–200 mm) and exclusion criteria (bilateral pitting edema; biologically implausible weight-for-height z-score (WHZ), weight-for-age z-score (WAZ), and height-for-age z-score (HAZ) values).

**Exposures:**

Weight was estimated using Broselow Tape, Hong Kong formula, and database MUAC alone, height alone, and height and MUAC combined.

**Main Outcomes and Measures:**

Mean percentage difference between true and estimated weight, proportion of estimates accurate to within ± 25% and ± 10% of true weight, weighted Kappa statistic, and Bland-Altman bias were reported as measures of tool accuracy. Standard deviation of mean percentage difference and Bland-Altman 95% limits of agreement were reported as measures of tool precision.

**Results:**

Database height was a more accurate and precise predictor of weight compared to Broselow Tape 2007 [B], Broselow Tape 2011 [A], and MUAC. Mean percentage difference between true and estimated weight was +0.49% (SD = 10.33%); proportion of estimates accurate to within ± 25% of true weight was 97.36% (95% CI 97.40%, 97.46%); and Bland-Altman bias and 95% limits of agreement were 0.05 kg and (-2.15 kg; 2.24 kg). The height model fitted for MUAC classes was accurate and precise. For MUAC < 115 mm, the proportion of estimates accurate to within ± 25% of true weight was 97.15% (95% CI 96.90%, 97.42%) and the Bland-Altman bias and 95% limits of agreement were 0.08 kg and (-1.21 kg; 1.37 kg). For MUAC between 115 and 125 mm, the proportion of estimates accurate to within ± 25% of true weight was 98.93% (95% CI 98.82%, 99.03%) and Bland-Altman bias and 95% limits of agreement were 0.05 kg and (-1.15 kg; 1.24 kg). For MUAC > 125 mm, the proportion of estimates accurate to within ± 25% of true weight was 98.33% (95% CI 98.29%, 98.37%) and Bland-Altman bias and 95% limits of agreement were 0.05 kg and (-2.08 kg; 2.19 kg).

**Conclusions and Relevance:**

Models estimating weight from height alone and height with MUAC class in children aged 6–59 months in a database from low-to-middle income countries were more accurate and precise than previous weight estimation tools. A height-based weight estimation tape stratified according to MUAC classes is proposed for children aged 6–59 months in limited-resource settings.

## Introduction

Estimation of weight is essential for the resuscitation of critically ill or injured children when time and / or instruments to measure weight for medication dosing and equipment selection are unavailable. A simple, accurate, and precise anthropometric tool for rapid estimation of weight in children would be especially useful in low-to-middle income countries where nearly all global under-five mortality occurs, severe acute malnutrition (SAM) is a significant contributor in approximately one-third of under-five mortality, and a scale to measure weight may not be immediately available to first-response providers.[1–6]

Among a variety of methods which have been developed to estimate weight in children, the length-based resuscitation tape (i.e. Broselow^™^ Pediatric Emergency Tape) is the most widely used. The latest version of Broselow Tape (BT) (2011 Edition A) incorporates adjusted length-weight classes based on National Health and Nutrition Examination Survey (NHANES) data from the United States. A previous version of BT accurately estimated weight from length in children ≤ 25 kg in the United States; however, BT (i.e. including 2011 Edition A, 2007 Edition B, and earlier) is known to both overestimate and underestimate weight in other populations of children in both high income countries and low-to-middle income countries.[7–34] Specifically, BT has been shown to overestimate weight in malnourished children.[7,11,19,27] The Malawi Tape represents a modification of BT to accommodate regional differences in weight-for-height.[35–36] BT (2007 Edition B) has been adjusted in India to create a new pediatric weight estimation tool for its malnourished population.[7] In Sudan, BT performance in estimating weight in children has been studied in the context of categories of malnutrition based on mid-upper arm circumference (MUAC), an accurate and reliable measure of acute undernutrition.[8] Like the Devised Weight Estimation Method (DWEM), the PAWPER tape in South Africa adds an appraisal of “body habitus” (based on a general visual impression of thinness / fatness rather than using a specific anthropometric technique or specific morphological features) to a length-based estimation of weight in children.[11,37–38] The Mercy Method, a weight estimation tool derived from NHANES data in the United States and based on MUAC and humeral length, estimates weight in selected populations of children without acute malnutrition more accurately in Mali than in India.[28–31]

MUAC alone has been used to estimate weight in both children and adults.[39–47] In healthy Chinese children living in Hong Kong, a formula based on MUAC:

estimated weight=3 × (MUAC−10)

with MUAC measured in cm and weight estimated in kg was shown to be at least as accurate and precise as BT (1998 version) in estimating weight in school-age children but was neither accurate nor precise in pre-school children.[41]

The purpose of this study is to investigate the accuracy and precision of MUAC and height as tools to estimate weight in children under five years of age in low-to-middle income countries.

## Materials and Methods

### Surveys

This was a retrospective observational study. Data were collected in 560 nutritional anthropometric surveys over a 171 month period between August 1992 and October 2006. Surveys were performed in locations with high prevalence of both acute and chronic malnutrition due to war, prolonged civil unrest, poor public health environment, and poor food security. Survey locations and the number of surveys from each location were: Afghanistan—35; Albania—1; Angola—17; Burundi—15; Central African Republic—2; Cote d'Ivoire—3; Chad—32; Democratic Republic of Congo—33; Eritrea—2; Ethiopia (NOS)—45; Ethiopia (Somali Region)—8; Guinea—2; Haiti—30; Indonesia—1; Kenya—7; Liberia—31; Macedonia—1; Malawi—9; Mozambique—9; Myanmar—8; Nicaragua—2; Niger—4; Pakistan—9; Rwanda—13; Sierra Leone—38; Somalia—17; Sri-Lanka—3; Sudan (Darfur)—28; Sudan (North)—47; Sudan (South)—66; Tajikistan—5; Tanzania—6; Uganda—30; and Zambia—1.

Surveys were conducted by Action Contre La Faim (ACF); CONCERN Worldwide; Emergency Nutrition Coordination Unit (ENCU) Ethiopia; Food Security Assessment Unit (FSAU) Somalia; GOAL Ireland; Médicins Sans Frontières (MSF) Belgium; Médicins Sans Frontières (MSF) Holland; Médicins Sans Frontières (MSF) Spain; Save the Children (SC) United Kingdom; and Save the Children (SC) United States.

### Data Collection and Management

The data collection methodology was consistent across the 560 surveys.[48] A modified Expanded Program of Immunization (EPI) two-stage cluster survey design was used. Primary sampling units (PSU) or “clusters” were selected from exhaustive lists of potential PSUs (e.g. villages, townships, census enumeration areas) using population proportional sampling (PPS). A minimum of *m* = 30 PSUs were always selected. The mean overall survey sample size was *n* = 811 children meeting study eligibility criteria. Within-PSU samples were taken using the EPI proximity sampling method. A single household was selected at random and subsequent households were selected by their proximity to the first household. All eligible children (i.e. children aged 6 to 59 months inclusive) in sampled households were measured. Sampling within each cluster stopped when a fixed sample size (usually *n* = 30) had been met or exceeded. Weight and height measurements were subject to standardization using the method of Habicht.[49] Standing height was recorded in children with a standing height of ≥ 85 cm. Supine length was recorded in children with a standing height < 85 cm. The term “height” is used in this report to refer to both standing height and supine length.

No clinical data were used. These were not medical experiments involving human subjects and, as such, are exempt from the terms of the Declaration of Helsinki. Whenever possible, data were collected following ethical approval from locally responsible ethics committees. Some data were collected during complex emergencies when no locally responsible ethics committees were operating. In these cases ethical approval was granted solely by the institutional review bodies of the non-governmental organization (NGO) or United Nations organization (UNO) which collected the data. Permissions were sought and given by local ministries of health and, where appropriate, by local police departments and military / paramilitary commanders. Identifying data were collected for programmatic purposes (i.e. for recruitment of cases of acute malnutrition into appropriate therapeutic feeding programs) but this data was either not entered or was removed prior to data being made available for analysis. Participation in the surveys was voluntary. In all surveys, the consent procedure was approved. Children were not (and could not be) measured without the consent of their parents or guardians. Verbal informed consent was sought from the primary caregiver of the child. Written consent is almost never sought in these types of survey: it is usually not required; and levels of literacy are often low. The existence of the data is proof of consent.

Data from these surveys were concatenated and the following inclusion criteria applied: age between 6 months and 59 months (inclusive); weight ≤ 25 kg; and MUAC between 80 mm and 200 mm (inclusive). Children with bilateral pitting edema were excluded because the weight of retained fluid tends to mask what would otherwise be low weight.[40] The weight estimate required for therapeutic purposes is the “normal” body weight rather than the upwardly biased “normal” body weight plus the weight of retained fluid. The estimation methods presented in this report aim to provide the desired estimate of weight. It should be noted that edema is not well recognized in many clinical and survey contexts. It is likely, therefore, that edema exclusions were limited to grade ++ and grade +++ edema. Children with biologically implausible weight-for-height z-score (WHZ), weight-for-age z-score (WAZ), and height-for-age z-score (HAZ) values were also excluded according to WHO Child Growth Standards guidelines.[50]

Of 459,036 children in all survey datasets, a total of 453,990 children passed these inclusion and exclusion criteria.

### Data Management and Analysis

Data management and data analyses were performed using the R Language for Data Analysis and Graphics.[51]

### Broselow Tape

Weight was estimated using BT 2007 [B] and BT 2011 [A] to the nearest of the 26 BT weight classes (i.e. 3–36 kg) using measured height in the database.

### Mid-Upper Arm Circumference

Weight was estimated from measured MUAC in the database (recorded in mm) using a linear model:

$$estimated\;weight_{\;1}=\;\alpha_{1}+\beta_{1}\cdot MUAC$$

**Fig 1:**
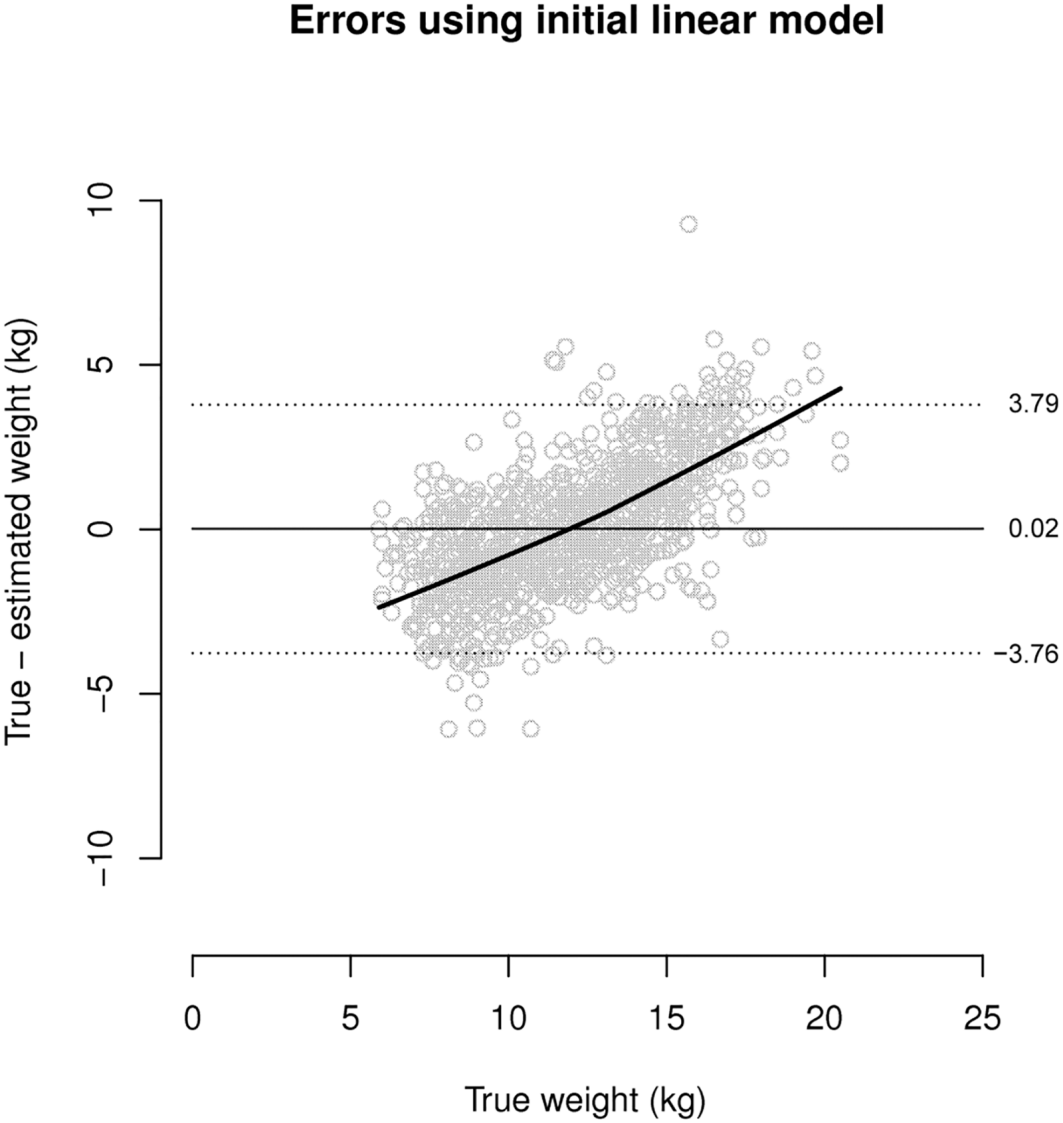
Pattern of errors found using a the initial linear model to estimate weight from MUAC (*n* = 897).

This model was fitted using a robust regression procedure.[52] Using this model to estimate weight from MUAC proved problematic. Examination of estimation errors revealed a marked systematic pattern. This pattern is illustrated in Fig 1 using data from a single survey from the Central African Republic (*n* = 897 children).

A correction was made by fitting a second linear model:

$$estimated\;weight_{\;1}=\alpha_{2}+\beta_{2}\cdot weight$$

and estimating weight as:

$$estimated\;weight=\bar{estimated\;weight_{\;1}}-\frac{\bar{estimated\;weight_{\;1}}-estimated\;weight_{\;1}}{\beta_{2}}$$

**Fig 2:**
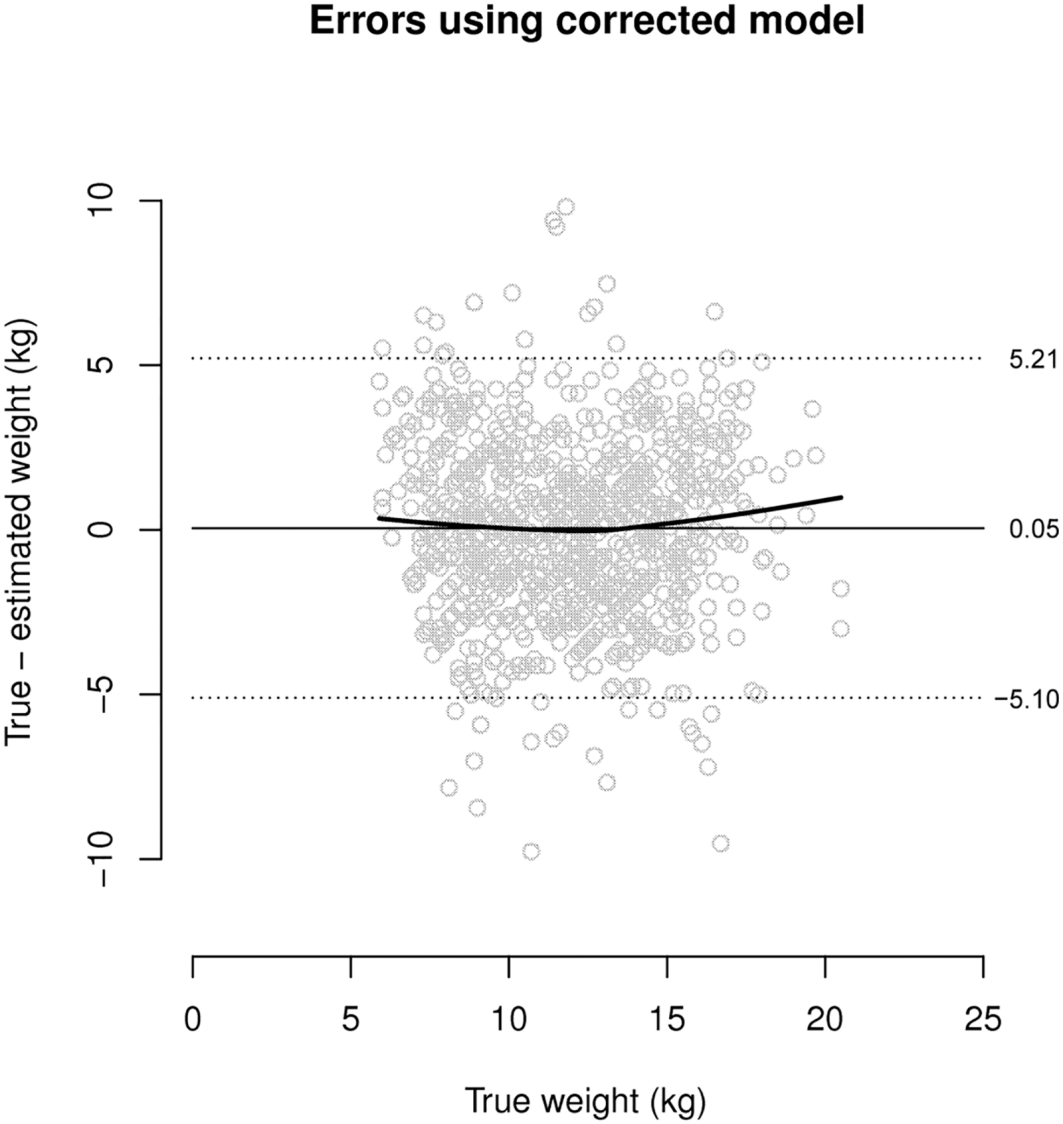
Pattern of errors found using the corrected (“rotated”) model to estimate weight from MUAC (*n* = 897).

This procedure removed the pattern of errors, as illustrated in Fig 2 which uses the same data as Fig 1. A corrected model was fitted using the complete dataset (*n* = 453,990 children). This model yielded the estimation formula:

$$estimated\;weight=11.2670-\frac{11.2670-\left(-9.0225+0.1445\cdot MUAC\right)}{0.4401}$$

for MUAC recorded in mm. We term the corrected model as having been “rotated” and call this model “MUAC1”.

Weight was also estimated from measured MUAC in the database (recorded in mm) using the Hong Kong formula.[41]

### Database Height / Length

Weight was estimated from measured height in the database using a linear rotated model (see above), which we call “HEIGHT1”, similar to that used for MUAC:

$$estimated\;weight=11.2967-\frac{11.2967-\left(-8.2993+0.2284\cdot height\right)}{0.8615}$$

### Stratification by MUAC

The HEIGHT1 model was fitted separately for three MUAC classes: MUAC < 115 mm corresponding to SAM; 115 ≤ MUAC < 125 mm corresponding to moderate acute malnutrition (MAM); and MUAC ≥ 125 mm corresponding to the absence of acute malnutrition. Weight was then predicted from height using the following estimation formulae:

$$estimated\;weight=7.0724-\frac{7.0724-\left(-5.8260+0.1769\cdot height\right)}{0.8196}$$

for SAM (i.e. MUAC < 115 mm);

$$estimated\;weight=8.2778-\frac{8.2778-\left(-5.5106+0.1802\cdot height\right)}{0.8779}$$

for MAM (i.e. 115 ≤ MUAC < 125 mm); and:

$$estimated\;weight=11.7344-\frac{11.7344-\left(-7.4675+0.2202\cdot height\right)}{0.8537}$$

for normal MUAC (i.e. MUAC ≥ 125 mm).

Testing these formulae for (e.g.) height = 90 cm gave weight = 10.76 kg for a child with SAM; weight = 11.05 kg for a child with MAM; and weight = 12.46 kg for a normal child. The model is well-behaved: weight estimated for the same height increases with increasing MUAC and the weight estimates for normal children approximate the WHO Child Growth Standards reference median (i.e. at 90 cm this is 12.6 kg for girls 2–5 years of age and 12.9 kg for boys 2–5 years of age).[50]

### Conversion to Weight Classes

Each model was adapted to yield narrow weight classes similar to those used by BT by solving the appropriate estimating formula for whole kg weights between 2 kg and 25 kg. We call these models “HEIGHT2” for height only and “HEIGHT3” for height and MUAC.

## Results

**Fig 3:**
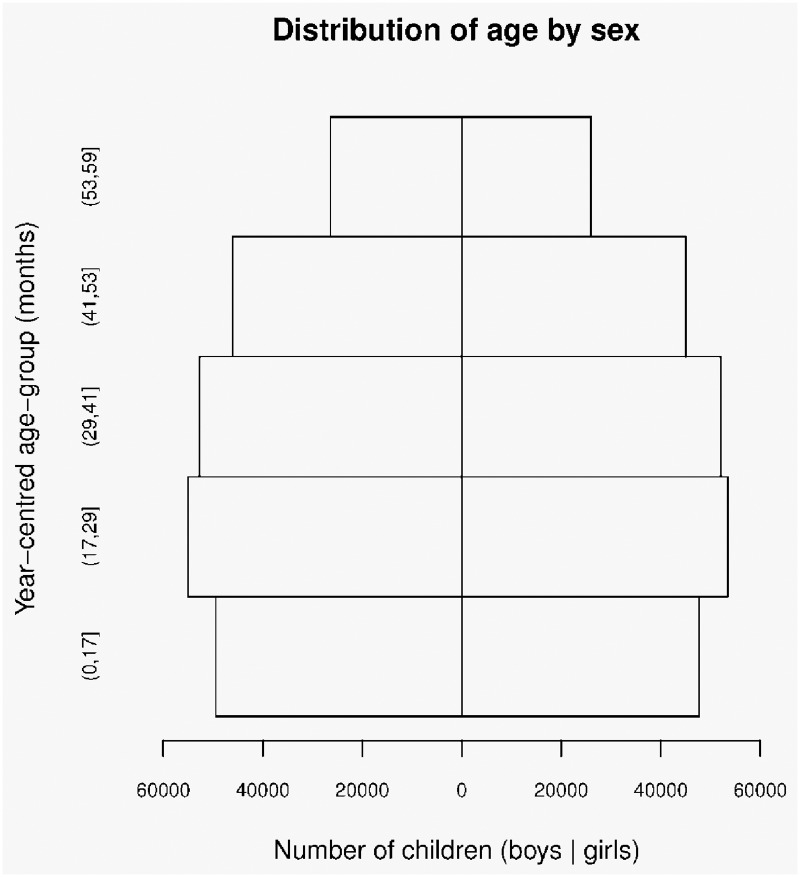
Distribution of age by sex in the study population (*n* = 453,990).

**Fig 4:**
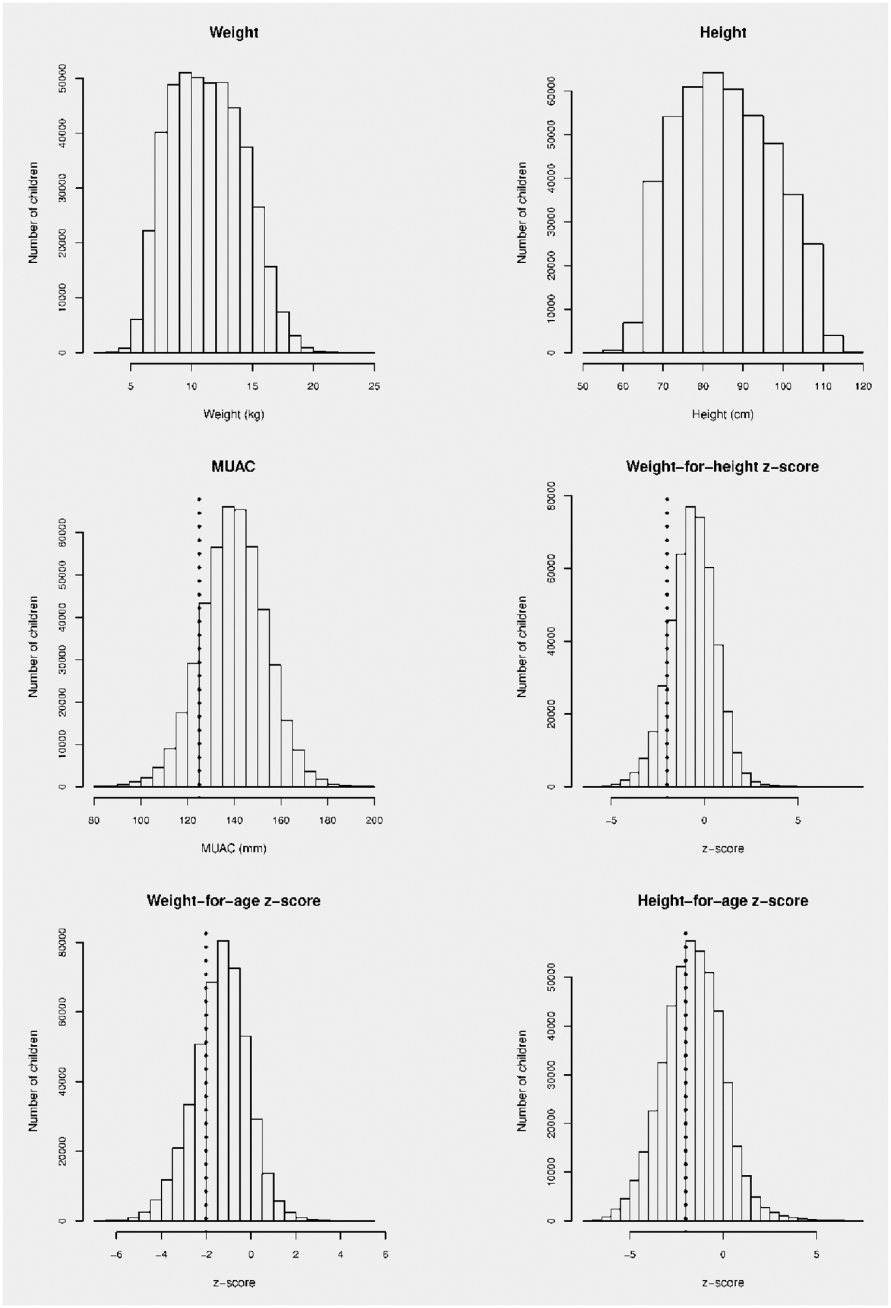
Distributions of selected anthropometric measures in the study population (*n* = 453,990).

Characteristics of the study population are shown in Table 1. Fig 3 shows the distribution of age by sex in the study population. Year-centered age-groups have been used in Table 1 and Fig 3 since considerable clustering of reported age at whole years and at 6 and 18 months was observed.[53] The distributions of weight, height, MUAC, WHZ, WAZ, and HAZ in the study population are shown in Fig 4. WHZ, WAZ, and HAZ were calculated using WHO Child Growth Standards data using purpose-written scripts.[50]

**Table 1:** Demographic and anthropometric characteristics of the study population (*n* = 453,990).

Variable	Summary	Class	Male	Female	Overall
Sex	n (%)	NA	229,615 (50.58)	224,375 (49.42)	453,990 (100.00)
Age class (months)*	mean (SD)	NA	32.29 (15.56)	32.40 (15.51)	32.35 (15.53)
	n (%)	(0,17]	49,432 (21.53)	47,671 (21.25)	97,103 (21.39)
		(17,29]	55,014 (23.96)	53,438 (23.82)	108,452 (23.89)
		(29,41]	52,699 (22.95)	52,126 (23.23)	104,825 (23.09)
		(41,53]	46,036 (20.05)	45,085 (20.09)	91,121 (20.07)
		(53,49]	26,434 (11.51)	26,055 (11.61)	52,489 (11.56)
Weight (kg)	mean (SD)	NA	11.5 (3.0)	11.2 (3.0)	11.3 (3.0)
Height (cm)	mean (SD)	NA	86.1 (11.9)	85.5 (12.1)	85.8 (12.0)
MUAC (mm)	mean (SD)	NA	140.85 (13.67)	140.03 (14.13)	140.44 (13.91)
Wasted by MUAC	n (%)	MUAC < 125	25,235 (10.99)	29,342 (13.08)	54,577 (12.02)
WHZ (z-score)	mean (SD)	NA	-0.68 (1.26)	-0.59 (1.18)	-0.64 (1.22)
Wasted by WHZ	n (%)	WHZ < -2 SD	32,516 (14.16)	24825 (11.06)	57,341 (12.63)
WAZ (z-score)	mean (SD)	NA	-1.44 (1.23)	-1.29 (1.19)	-1.36 (1.21)
Underweight by WAZ	n (%)	WAZ < -2 SD	69,722 (30.36)	57,125 (25.46)	12,6847 (27.94)
HAZ (z-score)	mean (SD)	NA	-1.72 (1.63)	-1.50 (1.58)	-1.61 (1.61)
Stunted by HAZ	n (%)	HAZ < -2 SD	98,388 (42.85)	83,529 (37.23)	18,1917 (40.07)

MUAC = mid-upper arm circumference; WHZ = weight-for-height z-score; WAZ = weight-for-age z-score; HAZ = height-for-age z-score; NA = not applicable

* Intervals (ranges) are expressed in ISO 31–11 form.[54] The form (a,b] expresses the interval a < x ≤ b. For example, (17,29] is used to indicate the set {18, 19, 20, 21, 22, 23, 24, 25, 26, 27, 28, 29} of ages in months.

Mean percentage difference between true weight and estimated weight (with SD percentage difference) are compared in Table 2 for the BT 2007 [B], BT 2011 [A], MUAC [Hong Kong formula], MUAC1, and HEIGHT1 weight estimation tools. Confusion matrices based on BT weight classes for both true and estimated weight were produced for all weight estimation tools and summary statistics (i.e. the proportion accurate to within ± 25% of true weight, the proportion accurate to within ± 10% of true weight, the proportion in the same BT weight class, the proportion accurate to within ± 1 BT weight class, weighted Kappa, and Bland-Altman bias and 95% limits of agreement) calculated. Results of this analysis are presented in Table 2.

**Table 2:** Comparison of weight estimation methods (*n* = 453,990).

Variable			BT 2007 [B]	BT 2011 [A]	MUAC [Hong Kong]	MUAC1	HEIGHT1
			Percentage Difference between True Weight and Estimated Weight Mean (SD)*				
**Weight (kg)**	(0,25]	All children	-4.58 (10.38)	-8.95 (11.24)	-7.68 (27.70)	+0.62 (29.92)	+0.49 (10.33)
	(0,10]	Children ≤ 10 kg	-9.64 (11.44)	-13.15 (11.12)	-11.48 (34.95)	+2.53 (39.08)	+1.55 (11.05)
	(10,25]	Children > 10 kg	-1.82 (9.12)	-6.54 (10.40)	-5.71 (24.25)	-0.16 (25.90)	-0.10 (9.88)
**MUAC (mm)**	< 115	Severely wasted	-22.76 (12.34)	-27.14 (12.36)	+64.27 (21.10)	+88.84 (20.79)	-9.39 (12.02)
	115 ≤ MUAC < 125	Moderately wasted	-15.46 (9.74)	-19.48 (10.25)	+24.18 (17.60)	+42.30 (16.16)	-4.87 (10.98)
	MUAC ≥ 125	Normal	-3.12 (9.76)	-7.46 (10.23)	-12.41 (25.17)	-5.02 (26.36)	-1.30 (9.99)
**WHZ**	WHZ < -3	Severely wasted	-31.08 (8.06)	-36.81 (7.73)	+33.96 (39.70)	+53.85 (45.89)	-21.63 (10.58)
	-3 < WHZ < -2	Moderately wasted	-20.05 (5.50)	-25.45 (5.29)	+15.13 (26.20)	+29.17 (28.39)	-13.27 (7.56)
	WHZ ≥ -2	Normal	-2.48 (9.09)	-6.69 (9.79)	-10.91 (26.07)	-3.31 (27.70)	2.34 (9.26)
**HAZ**	HAZ < -3	Severely stunted	-5.11 (11.47)	-8.54 (12.02)	-5.80 (30.28)	+5.52 (34.61)	3.01 (9.98)
	-3 < HAZ < -2	Moderately stunted	-3.92 (10.85)	-7.58 (10.59)	-10.15 (26.59)	-1.26 (29.56)	1.82 (9.61)
	HAZ ≥ -2	Normal	-4.67 (10.25)	-9.55 (11.01)	-7.30 (27.13)	0.00 (28.61)	-0.78 (10.46)
**WAZ**	WAZ < -3	Severely underweight	-\8.07 (10.90)	-22.00 (10.99)	+15.39 (34.42)	+32.74 (39.25)	-7.44 (10.39)
	-3 < WAZ < -2	Moderately underweight	-10.67 (9.00)	-14.55 (9.78)	-1.56 (26.50)	+10.16 (28.71)	-3.56 (9.74)
	WAZ ≥ -2	Normal	-1.56 (9.06)	-6.01 (9.93)	-11.64 (26.41)	-4.90 (27.76)	+2.48 (9.82)
Estimates accurate to within ± 25% of true weight**			95.58%	91.36%	61.78%	59.35%	97.36%
			(95.52%; 95.64%)	(91.28%; 91.44%)	(61.64%; 61.93%)	(59.21%; 59.49%)	(97.40%; 97.46%)
Estimates accurate to within ± 10% of true weight**			62.93%	51.67%	27.89%	26.25%	66.19%
			(62.79%; 63.08%)	(51.53%; 51.81%)	(27.77%; 28.03%)	(26.14%; 26.37%)	(66.05%; 66.32%)
Estimates in same BT weight class**^†^			64.83%	61.53%	30.37%	28.43%	63.23%
			(64.69%; 64.97%)	(61.39%; 61.67%)	(30.24%; 30.50%)	(28.30%; 28.56%)	(63.09%; 63.37%)
Estimates accurate to within ± 1 BT weight class**^†^			99.23%	98.94%	76.43%	73.00%	98.92%
			(99.20%; 99.26%)	(98.91%; 98.97%)	(76.31%; 76.55%)	(72.87%; 73.13%)	(98.89%; 98.94%)
Weighted Kappa**			0.8835	0.8756	0.6048	0.5922	0.8884
			(0.8807; 0.8863)	(0.8727; 0.8784)	(0.6030; 0.6066)	(0.5905; 0.5940)	(0.8856; 0.8913)
Bland-Altman bias and 95% limits of agreement^‡^			-0.44 (-2.47; 1.59)	-0.93 (-3.25;1.40)	-0.80 (-6.90; 5.30)	0.068 (-6.45; 6.59)	0.05 (-2.15; 2.24)

^Calculated as: $percentage\;difference\;=\;\frac{true\;weight-estimated\;weight}{true\;weight}\;\times \;100$

* Mean and SD were estimated using Huber M estimators of location and scale.[52] The mean percentage difference is a measure of systematic bias or accuracy. The SD percentage difference is a measure of precision. The difference in accuracy between any pair of methods can be assessed using the ratio of the absolute values of their mean percentage difference. The difference in precision between any pair of methods can be assessed using the ratio of their SD percentage differences. For example, comparing BT 2011 (A) and HEIGHT 1 in all children: $\Delta accuracy=\;\frac{\left|-8.95\right|}{\left|0.49\right|}=18.27\;\times \;improvement;\;\Delta \text{precision}=\frac{11.24}{10.33}=1.09\;\;\times \;improvement$ Values above one indicate better performance. Values of one indicate no difference in performance. Values below one indicate worse performance.

** Point estimate and 95% confidence interval

^†^ Appropriate BT classes for BT 2007 [B] and BT 2011 [A]. BT 2011 [A] classes are used for MUAC1 and HEIGHT1 results.

^‡^ Bias (mean of true—estimated weight, or mean error) and 95% limits of agreement were calculated following the method of Bland & Altman[55]

Results of weight estimation by HEIGHT1 fitted separately for three MUAC classes are presented in Table 3. Results of weight estimation for models HEIGHT2 and HEIGHT3 which yield narrow weight classes (see Table 4) are presented in Table 5.

**Table 3:** Weight estimation by HEIGHT1 fitted for three MUAC classes (*n* = 453,990).

Variable			MUAC SEVERE (MUAC < 115 mm)	MUAC MODERATE (115 ≤ MUAC < 125 mm)	MUAC NORMAL (MUAC ≥ 125 mm)
			Percentage Difference between True Weight and Estimated Weight Mean (SD)		
**Weight (kg)**	(0,25]	All children	1.20 (9.56)	0.58 (7.72)	0.55 (9.60)
	(0,10]	Children ≤ 10 kg	1.13 (9.20)	0.59 (7.54)	2.04 (9.83)
	(10,25]	Children > 10 kg	3.24 (16.05)	0.65 (8.77)	-0.09 (9.46)
**MUAC (mm)**	< 115	Severely wasted	1.20 (9.56)	NA	NA
	115 ≤ MUAC < 125	Moderately wasted	NA	0.58 (7.72)	NA
	MUAC ≥ 125	Normal	NA	NA	0.55 (9.60)
**WHZ**	WHZ < -3	Severely wasted	-6.91 (7.40)	-4.16 (3.85)	-27.20 (5.65)
	-3 < WHZ < -2	Moderately wasted	2.43 (5.32)	5.20 (5.32)	-16.32 (4.50)
	WHZ ≥ -2	Normal	12.52 (6.72)	2.12 (7.29)	3.72 (8.85)
**HAZ**	HAZ < -3	Severely stunted	2.11 (9.09)	0.76 (7.46)	3.72 (8.65)
	-3 < HAZ < -2	Moderately stunted	1.09 (9.20)	-0.75 (7.73)	1.88 (8.86)
	HAZ ≥ -2	Normal	-0.08 (10.24)	-2.53 (7.12)	-0.75 (9.81)
**WAZ**	WAZ < -3	Severely underweight	-1.07 (8.85)	-2.53 (7.12)	-6.78 (9.40)
	-3 < WAZ < -2	Moderately underweight	3.30 (8.62)	0.32 (6.99)	-4.16 (9.00)
	WAZ ≥ -2	Normal	11.21 (11.79)	4.67 (7.62)	1.98 (9.26)
Estimates accurate to within ± 25% of true weight			97.15%	98.93%	98.33%
			(96.90%;97.42%)	(98.82%;99.03%)	(98.29%;98.37%)
Estimates accurate to within ± 10% of true weight			68.72%	79.96%	69.72%
			(67.92%;69.47%)	(79.97%;80.34%)	(69.57%;69.85%)
Estimates in same BT weight class			71.75%	74.49%	65.46%
			(70.99%;72.47%)	(74.06%;74.92%)	(65.32%;65.61%)
Estimates accurate to within ± 1 BT weight class			98.72%	99.35%	99.21%
			(98.51%;98.89%)	(99.26%;99.42%)	(99.19%;99.24%)
Weighted Kappa			0.8289	0.8569	0.8791
			(0.8223;0.8355)	(0.8536;0.8601)	(0.8783;0.8798)
Bland-Altman bias and 95% limits of agreement			0.08 (-1.21;1.37)	0.05 (-1.15;1.24)	0.05 (-2.08;2.19)

**Table 4:** Weight class by height and MUAC class.

Weight (kg)	Height Only (cm)		MUAC SEVERE (MUAC < 115 mm)		MUAC MODERATE (115 ≤ MUAC < 125 mm)		MUAC NORMAL (MUAC ≥ 125 mm)	
	LOW	HIGH	LOW	HIGH	LOW	HIGH	LOW	HIGH
2	48.8	52.6	47.1	51.7	43.5	48.4	47.5	51.4
3	52.7	56.4	51.8	56.4	48.5	53.2	51.5	55.3
4	56.5	60.2	56.5	61.0	53.3	58.1	55.4	59.2
5	60.3	63.9	61.1	65.6	58.2	63.0	59.3	63.0
6	64.0	67.7	65.7	70.3	63.1	67.9	63.1	66.9
7	67.8	71.5	70.4	74.9	68.0	72.7	67.0	70.8
8	71.6	75.2	75.0	79.5	72.8	77.6	70.9	74.7
9	75.3	79.0	79.6	84.2	77.7	82.5	74.8	78.5
10	79.1	82.8	84.3	88.8	82.6	87.3	78.6	82.4
11	82.9	86.6	88.9	93.4	87.4	92.2	82.5	86.3
12	86.7	90.3	93.5	98.1	92.3	97.1	86.4	90.2
13	90.4	94.1	98.2	102.7	97.2	102.0	90.3	94.0
14	94.2	97.9	102.8	107.3	102.1	106.7	94.1	97.9
15	98.0	101.7	107.4	112.0	106.8	111.7	98.0	101.8
16	101.8	105.4	112.1	116.6	111.8	116.6	101.9	105.7
17	105.5	109.2	116.7	121.2	116.7	121.4	105.8	109.6
18	109.3	113.0	121.3	125.9	121.5	126.3	109.7	113.4
19	113.1	116.7	126.0	130.5	126.4	131.2	113.5	117.3
20	116.8	120.5	130.6	135.1	131.3	136.1	117.4	121.2
21	120.6	124.3	135.2	139.8	136.2	140.9	121.3	125.1
22	124.4	128.1	139.9	144.4	141.0	145.8	125.2	128.9
23	128.2	131.8	144.5	149.0	145.9	150.7	129.0	132.8
24	131.9	135.5	149.1	153.7	150.8	155.5	132.9	136.7
25	135.6	139.4	153.8	158.3	155.6	160.4	136.8	140.6

Caution: Extrapolation should be limited (indicated by gray shading) to 15% above (i.e. 126.5 cm) and 15% below (i.e. 43 cm) database height and to the lower limit of database weight (i.e. 3 kg).

**Table 5:** Weight estimation by height only (HEIGHT2) and height + MUAC (HEIGHT3) (*n* = 453,990).

Variable			HEIGHT2 Height Only	HEIGHT3 Height + MUAC
			Percentage Difference between True Weight and Estimated Weight Mean (SD)	
**Weight (kg)**	(0,25]	All children	0.50 (10.70)	0.66 (9.85)
	(0,10]	Children ≤ 10 kg	1.74 (11.42)	1.77 (9.89)
	(10,25]	Children > 10 kg	-0.04 (10.13)	0.02 (9.72)
**MUAC (mm)**	< 115	Severely wasted	-9.35 (13.41)	1.51 (11.05)
	115 ≤ MUAC < 125	Moderately wasted	-4.74 (11.68)	0.80 (8.85)
	MUAC ≥ 125	Normal	1.40 (10.38)	0.62 (10.08)
**WHZ**	WHZ < -3	Severely wasted	-21.68 (11.32)	-14.73 (12.41)
	-3 < WHZ < -2	Moderately wasted	-13.16 (8.47)	-10.24 (10.48)
	WHZ ≥ -2	Normal	2.46 (9.98)	2.10 (9.20)
**HAZ**	HAZ < -3	Severely stunted	3.15 (10.56)	3.46 (9.36)
	-3 < HAZ < -2	Moderately stunted	1.96 (10.26)	1.86 (9.35)
	HAZ ≥ -2	Normal	-0.70 (10.70)	-0.67 (10.05)
**WAZ**	WAZ < -3	Severely underweight	-7.29 (10.59)	-3.99 (9.71)
	-3 < WAZ < -2	Moderately underweight	-3.47 (10.06)	-3.00 (9.54)
	WAZ ≥ -2	Normal	2.57 (10.35)	2.19 (9.79)
Estimates accurate to within ± 25% of true weight			97.11% (97.06%;97.16%)	98.10% (98.06%;98.14%)
Estimates accurate to within ± 10% of true weight			64.63% (64.49%;64.77%)	68.62% (68.48%;68.76%)
Estimates in same BT weight class			58.26% (58.12%;58.41%)	60.62% (60.48%;60.76%)
Estimates accurate to within ± 1 BT weight class			98.17% (98.13%;98.21%)	98.53% (98.49%;98.57%)
Weighted Kappa			0.8745 (0.8739;0.8752)	0.8826 (0.8820;0.8832)
Bland-Altman bias and 95% limits of agreement			0.06 (-2.27;2.38)	0.07 (-1.97;2.10)

## Discussion

This study compared coarse anthropometric tools for weight estimation using a large international database of children aged between 6 and 59 months living in low-to-middle income countries and at risk of being undernourished. Previous studies of weight estimation in children have largely been conducted in developed countries and their applicability to children in limited-resource settings with varying degrees of wasting and stunting is unknown.[13–16,18,20–22,24–26,41–44,30–32,34,37–38] In these settings, a suitable scale to measure weight may not be available. Hence a tool for weight estimation would be useful for prescribing medications and selecting appropriately-sized equipment in emergencies. We looked at previously investigated anthropometric tools for weight estimation in children (i.e. height and MUAC) and found superior performance of height compared to MUAC.[7–26,41–47]

Ideal characteristics of a tool used for weight estimation, especially when needed urgently during resuscitation, are simplicity, accuracy, and precision. Simplicity is the consequence of the complexity of the required measurement / estimate and the design of a measurement / estimation tool. Accuracy of a measurement / estimation tool is the degree of nearness of a measurement / estimate to the true value. In this study, mean percentage difference between true weight and estimated weight, estimates accurate to within ± 25% and ± 10% of true weight, the weighted Kappa statistic, and Bland-Altman bias (i.e. the mean of true—estimated weight) were used as measures of tool accuracy.[55–58] Precision of a measurement / estimation tool is the degree of reproducibility of repeated measurements / estimates. In this study, the SD percentage difference and Bland-Altman 95% limits of agreement were used as measures of tool precision.[55]

BT is a tool for rapid weight estimation using weight classes based on the linear height-weight correlation. Wide use of BT in developed countries is based historically on its simplicity and accuracy. To date, BT has been shown to be accurate and precise only in children weighing < 10 kg when tested in low-to-middle income countries, but data are limited.[7–11,19] We tested both BT 2007 [B] and BT 2011 [A] “virtually” using the international database and found that BT 2007 [B] was more accurate and precise compared to BT 2011 [A] as a weight estimation tool for children living in low-to-middle income countries (Table 2). In children weighing 0 to 10 kg, both BT 2007 [B] and BT 2011 [A] tested using the international database were neither as accurate compared to the BT 2007 [B] tested in the United States nor as accurate and precise as the most recent version of BT tested in India in 2006 (Table 2).[18–19] Of note in children weighing 10 to 25 kg, BT 2007 [B] was a more accurate weight estimation tool when tested against the large international database compared to a smaller database from the United States (Table 2).[18]

Existing studies have shown variable potential for MUAC as a weight estimation tool in children, but MUAC has not been fully tested in low-to-middle income countries where it is already used widely as a measure of acute undernutrition.[41–47] A linear model was initially used to estimate weight from MUAC, but due to a significant pattern of inaccuracy (i.e. overestimation in children of lower weight and underestimation in heavier children) was replaced with a corrected, or “rotated”, linear model. When the corrected model was tested using the entire international database, MUAC1 was found to lack precision as a weight estimation tool (Table 2). Compared to BT, MUAC1 was more accurate by mean percentage difference and Bland-Altman bias, but less precise (Table 2). MUAC tested by the Hong Kong formula lacked both accuracy and precision as a weight estimation tool (Table 2).

Because of the potential and familiarity of the linear height-weight relationship as a tool for weight estimation, the next step was to investigate a linear model to estimate weight directly from measured height in the international database. Specifically, we were interested to determine which height-based tool would perform best in estimating weight: the database-derived linear model or BT. After the initial linear model was corrected to reduce error, we found the linear rotated model HEIGHT1 to be: more accurate than either BT or MUAC alone; more precise than MUAC alone; and of similar precision to BT (Table 2). A simple linear tape based on HEIGHT1 was considered for field use to estimate weight in children. However we noted that the accuracy of HEIGHT1 model as a weight estimation tool deteriorated according to severity of malnutrition measured by MUAC, WHZ, WAZ, and HAZ (Table 2). We then fitted HEIGHT1 for three separate MUAC classes (i.e. SAM, MAM, absence of acute malnutrition) and observed improved accuracy and precision compared to HEIGHT1 (Table 3). The fitted model was then converted to two additional models based on sequential 1 kg wide weight classes (i.e. HEIGHT2 for height only and HEIGHT3 for height and MUAC class) without loss of accuracy or precision (Table 5). The weight classes which are shown in Table 4 could be used to produce a height-to-weight tape similar to BT but with stratification according to nutritional status defined by MUAC class.

The study had several potential limitations. Firstly, testing of BT was virtual. We are unaware if this would lead to significantly different outcomes of accuracy and precision compared to live testing. Secondly, the results of this study are applicable only to children with age between 6 and 59 months, weight between 2 and 25 kg, and with height between 49 and 137.5 cm. While the scope of children covered by the study is limited, the database which was available for this study does match the age range (i.e. between 6 and 59 months) of the recent United Nations Millennium Development Goal 4 (two-thirds reduction of U5M from 1990 levels by 2015). It is hoped that a weight estimation tool might assist health care workers in low-to-middle income countries who work in pediatric health maintenance clinics and respond to child health emergencies. Thirdly, the study population represents an incomplete sample of children with SAM. Diagnostic indicators for SAM in children aged between 6 and 59 months include: severe wasting (i.e. MUAC < 115 mm; WHZ < -3 SD); and bilateral pitting edema.[59] The study included children with MUAC 80–200 mm but excluded those with bilateral pitting edema because weight is overestimated in these children. Fourthly, we reported estimates accurate to within ± 10% and ± 25% of true weight as measures of tool accuracy. Our choices were based on the general rule-of-thumb in routine practice that an accuracy of 10–20% for dosing of fluids and most drugs during resuscitation is reasonable.[60] We acknowledge that the clinical significance of making weight errors in children has not been well established.

## Conclusions

We found a model which estimated weight directly from database height to be more accurate and precise compared to BT or MUAC. A simple height-based weight estimation tape stratified according to MUAC is proposed for children aged 6–59 months in limited-resource settings.
